# Harnessing dual variational autoencoders to decode microbe roles in diseases for traditional medicine discovery

**DOI:** 10.3389/fphar.2025.1578140

**Published:** 2025-05-30

**Authors:** Qing Ye, Yaxin Sun

**Affiliations:** ^1^ School of Data Science and Artificial Intelligence, Wenzhou University of Technology, Wenzhou, China; ^2^ School of Computer Science and Technology (School of Artificial Intelligence), Zhejiang Normal University, Jinhua, China; ^3^ Department of Algorithm, Zhejiang Aerospace Hengjia Data Technology Co., Ltd., Jiaxing, China

**Keywords:** traditional medicine discovery, microbe-disease association, double variational autoencoders, multi-information fusion, prediction model

## Abstract

Traditional medicine encompasses a rich trove of knowledge and practices for disease prevention, diagnosis, and treatment. However, it faces challenges such as poorly defined compositions of preparations and limited high-quality efficacy data. The development of artificial intelligence presents new opportunities for traditional medicine research and applications, especially in predicting MDAs (MDAs), which is of great significance for understanding disease mechanisms and developing new treatments. This study proposes a MDAs prediction method based on double variational autoencoders (DVAMDA). This method innovatively integrates double variational autoencoders and multi-information fusion techniques. Firstly, the graph SAGE encoder is utilized to preliminarily extract the local and global structural information of nodes. Subsequently, the double variational autoencoders are employed to separately extract the latent probability distribution information of the initial input data and the graph-specific property information from the output of the graph SAGE encoder. Then, these different sources of information are fused to provide rich and powerful feature support for subsequent prediction tasks. Finally, the Hadamard product operation and a deep neural network are used to predict MDAs. Experimental results on the HMDAD and Disbiome datasets show that the DVAMDA model performs outstandingly in multiple evaluation metrics. The findings of this research contribute to a deeper understanding of microbe-disease relationships and provide strong support for drug development in traditional medicine based on MDAs. The relevant data and code are publicly accessible at: https://github.com/yxsun25/DVAMDA.

## 1 Introduction

As a core component of humanity’s medical treasure trove, traditional medicine has provided a rich source of innovative inspiration for modern drug discovery through its thousands of years of accumulated experience in disease prevention and treatment. However, its modernization faces severe challenges: the ambiguity of complex formulas, fragmentation of efficacy data, and complexity of microbe-disease association mechanisms have made it difficult to precisely target key biological targets in traditional drug discovery. In recent years, breakthroughs in artificial intelligence (AI) have opened new avenues to address these challenges-by efficiently processing high-dimensional biological data and uncovering latent associations, AI can deeply decode the complex relationships between microbial communities and disease phenotypes, serving as a bridge between traditional medical experience and modern precision medicine. Among these, predicting microbe-disease associations (MDAs), a critical step in understanding disease mechanisms and identifying drug intervention targets, has emerged as a frontier direction for AI-empowered traditional drug discovery. While existing methods have made progress in MDA prediction. These methods can be divided into three categories: matrix factorization-based methods, link propagation-based methods, and deep learning-based methods.

Matrix factorization-based approaches have been pivotal in predicting MDAs. A low-rank sparse matrix completion is used to augment the association (Yu et al., 2024). A heterogeneous network is constructed by disease and microbe similarities, and then simplified the MDA prediction task into a low-rank matrix completion problem (Liu Haiyan et al., 2023). The weighted k-nearest neighbor algorithm and cross-domain matrix factorization is used to optimize the association matrix for prediction (Chen Jing et al., 2024). The generalized matrix factorization based on weighted hypergraph learning is designed to enhance the prediction (Ma and Liu, 2022). Low-rank representation is used to fuse multiple similarity information to capture more comprehensive features (Liu Jin-Xing et al., 2023). Microbe-disease similarities and known associations are integrated to build a heterogeneous network (Yan et al., 2021). Multiple similarity measures are used to construct Kronecker similarities (Xu et al., 2021). Matrix factorization-based methods play a significant role in MDA prediction. However, they also face challenges, such as dealing with complex data and improving computational efficiency. These methods often struggle when the data has high-dimensionality or complex non-linear relationships. The computational cost of matrix operations, especially in large-scale datasets, can be prohibitively high, limiting their practical application in real - time or resource-constrained scenarios.

Link propagation-based methods provide an alternative way to predict MDAs. Network topological similarity is used to constructed a heterogeneous network and assigns its weights (Luo and Long, 2020). A computational model of node-information-based link propagation is designed for MDAs (Peng Li et al., 2020). PU learning is used to select reliable negative samples and the Mahalanobis distance function with the k-nearest neighbor algorithm is used for prediction (Wang Jianru et al., 2023). Multiple similarities are integrated by two-tier bi-random walk and the weighted k-nearest neighbor algorithm is used for prediction (Yin et al., 2023). A bi-directional heterogeneous microbial disease network is constructed by integrating multiple similarities, including Gaussian kernel similarity, microbial function similarity, disease semantic similarity, and disease symptom similarity and the neighbor information of the network is learned by random walk (Guan et al., 2022). A new method is designed by integrating multiple data sources and path-based heteSim scores (Fan et al., 2019). A linear neighborhood label propagation with multi-order similarity fusion learning is proposed for MDA perdition (Chen Ruibin et al., 2024). Microbe and disease networks with known associations are integrated to calculate similarities (Yan et al., 2020). Network consistency projection and label propagation are used together for MDA (Yin et al., 2022). These methods leverage different network-based techniques and algorithms to effectively predict MDA, but they also face challenges like handling noisy data and improving prediction accuracy in complex scenarios. Noisy data can lead to inaccurate similarity calculations and network construction, thus affecting the prediction results. In complex scenarios with many interacting factors, it becomes difficult to accurately capture and model all the relationships, resulting in lower prediction accuracy.

Deep learning-based methods have revolutionized the field of predicting MDAs, offering advanced techniques to handle complex data and capture intricate relationships. These methods can be broadly classified into two categories: those centered around neural network-based feature extraction and classification, and those leveraging graph-based deep learning architectures.

Some methods focus on using neural networks to extract features. A deep sparse autoencoder neural network is used to extract effective features from the data and then a random forest classifier is used to predict potential MDA (Wang Liugen et al., 2023). A non-negative matrix tri-factorization model, bi-random walk model, and capsule neural network are used together to extract features from different perspectives (Peng et al., 2023). A specific activation function and an initial weight optimizing method are used to improve the training speed and prediction accuracy (Li et al., 2021). The disease and microbe one-hot encodings are fed into neural network, which is transformed into a low-dimensional dense vector in implicit semantic space via embedding layer (Liu et al., 2021). A large-scale information network embedding is designed for network embedding (Wang et al., 2022). A dual network contrastive learning is used to learn the representation (Cheng et al., 2023). A text mining framework based on a pretrained model BERE is designed for microbe-disease interaction extraction (Wu et al., 2021). A multi-view multi-modal network and a multi-scale feature fusion mechanism are used together (Wang Shuang et al., 2024). These methods centered around neural network-based feature extraction and classification have shown great potential. However, they may suffer from issues such as overfitting, especially when the training data is limited. Additionally, the interpretability of these models is often poor, making it difficult to understand how they arrive at their predictions, which can be a significant drawback in medical applications where explainability is crucial.

Microbe-disease relationships are typically graph-structured data, and many graph-based deep learning methods have been designed. An autoencoder and adversarial regularization mechanism is used to learn node representations (He et al., 2024). Graph convolutional networks are used to learn the embeddings of diseases and microbes that integrates various associations and similarities (Wu et al., 2025). A graph attention network which contains components like a decomposer, combiner, and predictor is designed to effectively analyze and predict associations (Liu et al., 2022). A microbe-drug-disease tripartite network is constructed and furthered processed by a relation graph convolutional network (Wang Yueyue et al., 2023). Multiple layers of embedding features are learned by graph SAGE (Dai et al., 2023). A novel graph autoencoder framework that utilizes decoupled representation learning and multi-scale information fusion strategies to efficiently infer potential MDAs (Wang Wentao et al., 2024). Graph convolutional networks and multi-neighborhood graph convolutional networks are used for feature extraction and structure information capture (Chen and Chen, 2024). A higher-order graph attention network is used to extract the features for the node, and an inner product decoder is used to reconstruct the MDA matrix (Wang et al., 2022). A graph convolutional network to learn embeddings for diseases and microbes, and a score function is used for prediction (Jiang et al., 2023). Furthermore, deep sparse auto-encoder (Lu et al., 2023), graph variational autoencoders (Zhu et al., 2024), generative adversarial networks (Naik et al., 2024), multi-attention blocks (Hua et al., 2022) are leveraged in the graph-based deep learning architectures. These methods, while effective in capturing graph-related information, face challenges like high computational complexity due to the intricate graph operations. They may also be sensitive to the quality of the graph construction, and small errors in graph data can lead to significant deviations in prediction results. Moreover, similar to other deep learning models, their interpretability remains a challenge, which is a concern in medical research and diagnosis.

To overcome the numerous problems of the above - mentioned methods and further improve the prediction performance of MDAs, this paper elaborately designs a prediction method based on double variational autoencoders (DVAMDA). This method ingeniously integrates double variational autoencoders and multi-information fusion techniques to construct an efficient prediction framework. Specifically, the graph SAGE encoder is first used to preliminarily explore the local and global structural features of nodes, laying a foundation for subsequent analysis. Subsequently, through the uniquely designed double variational autoencoders, the latent probability distribution information is extracted from the initial input data, and the graph-specific property information, such as node connectivity and neighborhood relationships, is mined from the output of the graph SAGE encoder. Then, these three key types of information from different sources are fused, providing more in-depth and extensive feature support for subsequent analysis and prediction tasks. Finally, the Hadamard product operation and a deep neural network (DNN) are applied to accurately predict MDAs. Through this series of innovative designs, DVAMDA is expected to break through the limitations of traditional methods and achieve better results in the field of MDA prediction.

Overall, our contributions are summarized as follows:

(1) The double variational auto-encoders are pivotal. They extract latent probability distribution for both DNN-like features and graph-related characteristics at different levels. The use of different-level loss functions can guide the model to learn more meaningful features at different levels, train the model with limited samples and enable an increase in network depth.
(2) The multi-information fusion technique effectively combines various feature types. By integrating outputs from different components, DVAMDA comprehensively leverages the advantages of diverse feature sources.
(3) The sequential use of the Graph SAGE Encoder and GVAEs for graph feature extraction has unique benefits. The Graph SAGE Encoder captures the basic graph structure, and the GVAEs refine and expand on these features considering graph - specific properties.

## 2 Materials and methods

**FIGURE 1:**
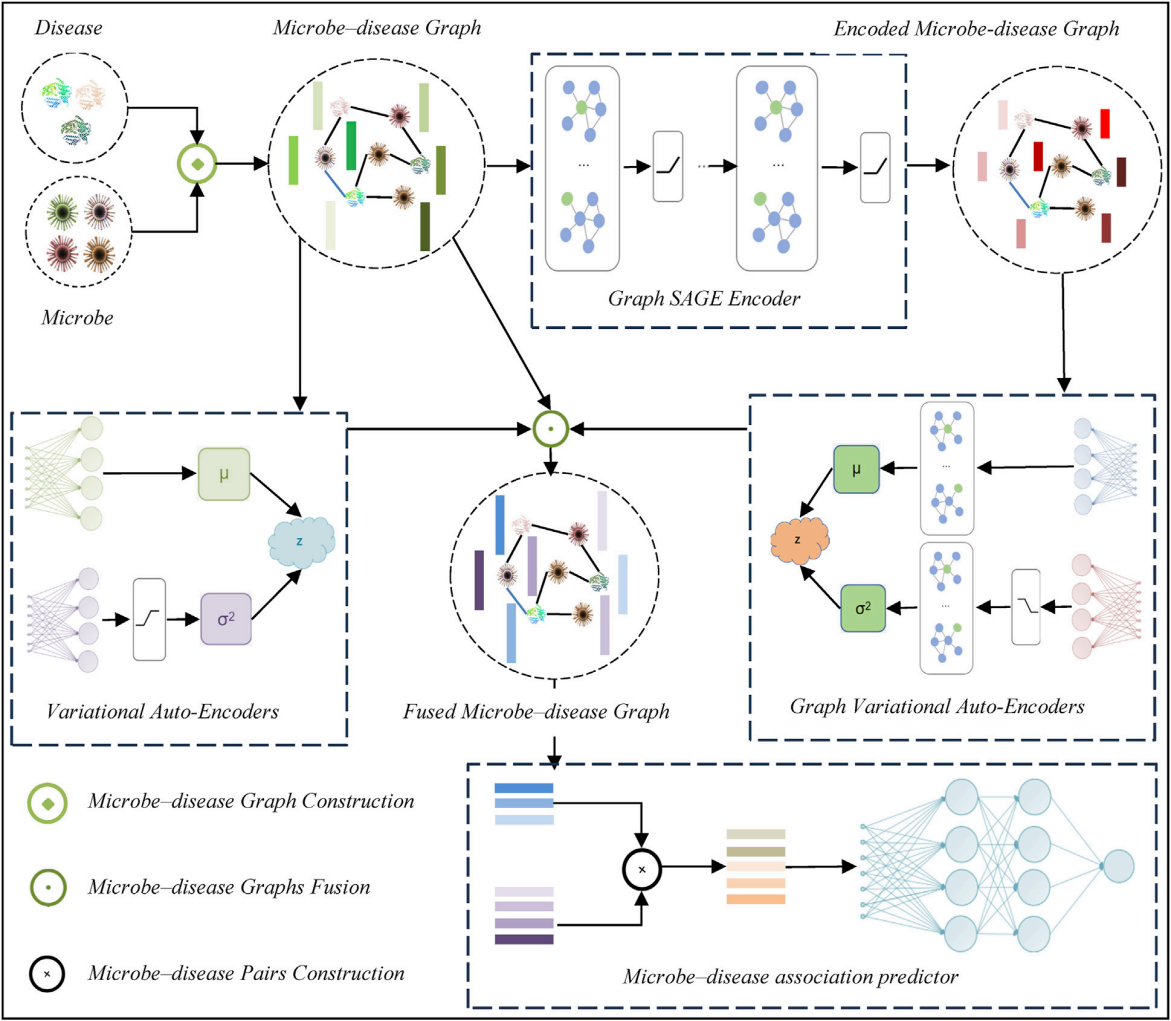
DVAMDA structure.

The proposed DVAMDA method for MDA prediction follows a multi-step framework, which is shown in Figure 1. Firstly, it constructs a microbe-disease graph to represent the relationships between microbes and diseases. Then, a Graph SAGE encoder is utilized to extract features from this graph. Next, variational auto-encoders and graph variational auto-encoders are employed to process and transform the features. After that, microbe-disease graphs are fused to integrate different aspects of information. Subsequently, microbe-disease pairs are constructed. Finally, an MDA predictor is used to predict the associations between microbes and diseases based on the processed data and features.

### 2.1 Microbe–disease graph construction

In this study, an in-depth exploration of MDAs was conducted. The data was predominantly sourced from two authoritative databases. The simple statistics for the sample information of these two MDAs datasets are shown in Table 1. The initial one is the HMDAD database, accessible via the URL http://www.cuilab.cn/hmdad. This database has been meticulously curated and encompasses 450 experimentally validated associations. These associations pertain to as many as 39 distinct diseases and 292 diverse microbes, as elaborated in reference (Ma et al., 2017). The experimentally validated associations are of paramount significance as they furnish reliable evidence for the correlation between microbes and diseases.

**TABLE 1:** Simple statistics for the sample information of Three MDAs datasets.

Data sets	Number of microbes	Number of diseases	Number of microbe- disease associations
HMDAD	292	39	450
Disbiome	1052	218	4351
MMDA	1177	134	4499

The Disbiome database, with its official website at https://disbiome.ugent.be/home, was also incorporated. The Disbiome database is even more comprehensive, accommodating a total of 4351 associations. These associations span across 218 different diseases and 1052 various microbes, as cited in reference (Janssens et al., 2018). The all-encompassing nature of the Disbiome database enriches the dataset and enables a more profound analysis.

The MMDA database (Chen Jing et al., 2024) is meticulously assembled by integrating data sourced from the HMDAD (Schriml et al., 2022), Disbiome (Long et al., 2021), and Peryton (Hua et al., 2022) databases. Prior to integration, a series of rigorous data preprocessing steps are implemented, including de-duplication, simplification, and removal of irrelevant items.

Additionally, semantic similarity features of diseases and functional similarity features of microbes were extracted from the disease ontology database (Schriml et al., 2022). By utilizing the microbe-disease association matrix, the Gaussian kernel similarity, cosine similarity, and SIGMOD functional kernel similarity for both microbes and diseases were calculated (Li et al., 2021). Given the MDA matrix $A\in R^{m\times n}$ , where *m* and *n* are numbers of microbes and diseases, features of microbes *M* can be calculated by Equation 1:

$$M=\left(GK+CS+SK\right)/3$$ (1)

Where GK , CS and SK represent the Gaussian kernel similarity, cosine similarity, and SIGMOD functional kernel similarity for microbes.

Similarly, features of diseases *D* can be calculated by Equation 2:

$$D=\left(GK+CS+SK\right)/3$$ (2)

As can be seen from Table 1, the number of microbes is larger than that of diseases. Therefore, the dimension of *M* is greater than that of *D*. To make the dimensions of the two consistent, the zero - padding method is used to pad the dimension of *D* to be the same as that of *M*, obtaining $D^{+}$ . Finally, $D^{+}$ and *M* are concatenated row-wise to derive the hypergraph node feature matrix *X* by Equation 3:

$$X=\left[M;D^{+}\right]$$ (3)

After obtaining the X , based on the MDA matrix, the observed microbe-disease graph was constructed and denoted as G=<V,E,X> . In this graph, V encompasses all microbes and diseases, E represents the set of observed MDAs, and X is defined as the initial node feature representation, which serves as the fundamental input for subsequent graph-based analysis and prediction tasks.

### 2.2 Graph SAGE encoder

In the task of MDA prediction, effectively extracting feature information from the constructed microbe-disease graph is of crucial importance. The graph sample and aggregate encoder (Graph SAGE encoder) (Hamilton et al., 2017) is a robust graph neural network encoder. Unlike traditional encoders that necessitate global training on the entire graph, graph SAGE learns node representations through sampling and aggregating the neighbors of nodes. This characteristic endows it with high efficiency and scalability when processing large-scale graph data. Given the large number of microbe and disease nodes and their intricate associated edges in the microbe-disease graph, graph SAGE is particularly well-suited for this task. Moreover, it can capture the local structural information of nodes, which is instrumental in uncovering the potential relationships between microbes and diseases. This, in turn, lays a solid foundation for subsequent feature processing and association prediction.

Given the constructed microbe-disease graph G=<V,E,X> , for each node v∈V , at the *l*th layer, the graph SAGE encoder updates its feature representation by aggregating the features of its neighbor nodes. The aggregation process (Jiang et al., 2023) can be expressed by Equation 4:

$$h_{v}^{l}=\sigma \left(W^{l}\cdot {AGGREGATE}_{l}\left(\left\{h_{u}^{l-1},\forall u\in N\left(v\right)\right\}\right)+W^{l}\cdot h_{v}^{l-1}\right)$$ (4)

Where $h_{v}^{l}$ is the feature vector of node v at the *l*th layer. $W^{l\;}$ is the trainable weight matrix at the *l*th layer. σ is an activation function, such as rectified linear unit (ReLU), etc., which is used to introduce non-linearity. ${AGGREGATE}_{l}\left(\cdot \right)$ is the aggregation function at the *l*th layer. Common aggregation functions include mean aggregation, long short-term memory aggregation, and pooling aggregation, etc (Hamilton et al., 2017). In this study, the mean aggregation is used, due to its high computational efficiency, ability to retain overall neighbor information, and smoothing effect on feature representation. the mean aggregation (Hamilton et al., 2017) can be calculated by Equation 5:

$${AGGREGATE}_{l}\left(\left\{h_{u}^{l-1},\forall u\in N\left(v\right)\right\}\right)=\frac{1}{\left|N\left(v\right)\right|}\sum_{u\in N\left(v\right)}h_{u}^{l-1}$$ (5)

After passing through multiple graph aggregation layers defined by Equation 1, the encoded microbe-disease graph $\tilde{G}=<V,E,\tilde{X}>$ can be obtained. Here, $\tilde{X}$ represents the mid-level features that possess graph-specific properties, including node connectivity and neighborhood relationships. The graph SAGE encoder is a typical graph neural network, and the features $\tilde{X}$ extracted by it can be directly utilized for MDA prediction. However, in the microbe-disease graph, the number of edges is relatively small, resulting in a sparse graph structure. This sparsity issue leads to a limited amount of training data. When dealing with such sparse graphs, setting a large number of layers in the graph SAGE encoder is not advisable. A deeper architecture may cause the model to focus too much on the limited training data, capturing noise and idiosyncrasies rather than the underlying patterns. As a consequence, the trained model is prone to overfitting, thus failing to generalize effectively in practical MDA prediction scenarios. To address these challenges, double variational auto-encoders are further employed for feature extraction.

### 2.3 Double variational auto-encoders

After passing through the graph SAGE encoder, there are the original microbe-disease graph G and the encoded microbe–disease graph $\tilde{G}$ . For these two types of data, variational auto-encoders (VAEs) and graph variational auto-encoders (GVAEs) are respectively employed for further processing.

VAEs are selected to handle the initial input due to their ability to learn a probabilistic representation of the data. In the context of MDA prediction, the initial input data typically contains complex and noisy information. Microbial and disease-related data can be affected by numerous factors, such as environmental variables, genetic diversities, and experimental inaccuracies. VAEs can effectively model the distribution of this data in a latent space. This latent space representation enables better handling of the uncertainty and variability present in the data. By mapping the input data to the latent space, VAEs can capture the underlying patterns and structures related to microbes and diseases. This probabilistic representation is valuable as it can provide insights into the relationships between different data points and assist in generating new data points similar to the original ones, which can be useful for predicting novel MDAs.

The encoding process of a VAE maps the input data x∈X to a posterior distribution $q_{\emptyset }\left(Z|X\right)$ over the latent variables Z , where ∅ represents the parameters of the encoder (Diederik and Welling, 2019). Typically, the encoder approximates the posterior distribution (Diederik and Welling, 2019) as a Gaussian distribution with mean μ and variance $\sigma^{2}$ , as specified by Equation 6:

$$q_{\emptyset }\left(Z|X\right)=Ν\left(z;\mu_{\emptyset }\left(X\right),\sigma_{\emptyset }^{2}\left(X\right)\right)$$ (6)

The mean $\mu_{\emptyset }\left(X\right)$ and variance $\sigma_{\emptyset }^{2}\left(X\right)$ are calculated by Equations 7, 8:

$$\mu_{\emptyset }\left(X\right)={Encoder}_{\emptyset }\left(X\right)$$ (7)

$$\sigma_{\emptyset }^{2}\left(X\right)={Encoder}_{\emptyset }^{\prime }\;\left(X\right)$$ (8)

Here, ${Encoder}_{\emptyset }$ and ${Encoder}_{\emptyset }^{\prime }$ are neural network functions parameterized by ∅ . Given that the sample size of MDA data is relatively small, and the purpose of the double variational auto-encoders is to extract features at different levels, the ${Encoder}_{\emptyset }$ only consists of a single linear layer, and the ${Encoder}_{\emptyset }^{\prime }$ only includes a linear layer followed by a normalization layer, as shown in Figure 1. The limited sample size restricts the complexity of the encoder architecture to avoid overfitting. A simple linear layer in ${Encoder}_{\emptyset }$ can capture the basic linear relationships in the data. The addition of a normalization layer in ${Encoder}_{\emptyset }^{\prime }$ helps to standardize the output, making the variance calculation more stable and facilitating better training of the VAE model.

After the graph SAGE encoder processes the microbe-disease graph and generates node embeddings, GVAEs are utilized. GVAEs are specifically designed to handle graph-structured data within a variational framework. The node embeddings obtained from the graph SAGE encoder encapsulate both the local and global structural information of the graph within a feature space. GVAEs have the ability to further model the distribution of these embeddings in a latent space while simultaneously taking into account graph-specific properties such as node connectivity and neighborhood relationships. Node connectivity determines how closely related different nodes are in the graph, and neighborhood relationships describe the immediate and extended surroundings of each node. By considering these properties, GVAEs can extract more refined and graph-aware latent representations. These representations are of utmost importance for accurately predicting MDAs as they are capable of capturing the complex topological and relational information present in the graph.

Given the $\tilde{G}=<V,E,\tilde{X}>$ obtained by graph SAGE encoder, the encoder of a GVAE maps the $\tilde{G}$ to a posterior distribution ${\tilde{q}}_{\tilde{\emptyset }}\left(\tilde{Z}|\tilde{G}\right)$ over the latent node embeddings $\tilde{Z}$ . Similar to VAEs, it can be modeled as a Gaussian distribution with mean $\tilde{\mu }$ and variance ${\tilde{\sigma }}^{2}$ , as defined by Equation 9.

$${\tilde{q}}_{\tilde{\emptyset }}\left(\tilde{Z}|\tilde{G}\right)=Ν\left(\tilde{Z};{\tilde{\mu }}_{\tilde{\emptyset }}\left(\tilde{G}\right),{\tilde{\sigma }}_{\tilde{\emptyset }}^{2}\left(\tilde{G}\right)\right)$$ (9)

The mean ${\tilde{\mu }}_{\tilde{\emptyset }}\left(\tilde{G}\right)$ and variance ${\tilde{\sigma }}_{\tilde{\emptyset }}^{2}\left(\tilde{G}\right)$ are calculated using two graph-neural network-based encoders, represented by Equations 10, 11, respectively.

$${\tilde{\mu }}_{\tilde{\emptyset }}\left(\tilde{G}\right)={GNNEncoder}_{\tilde{\emptyset }}\left(\tilde{G}\right)$$ (10)

$${\tilde{\sigma }}_{\tilde{\emptyset }}^{2}\left(\tilde{G}\right)={GNNEncoder}_{\tilde{\emptyset }}^{\prime }\;\left(\tilde{G}\right)$$ (11)

Where ${GNNEncoder}_{\tilde{\emptyset }}$ and ${GNNEncoder}_{\tilde{\emptyset }}^{\prime }$ are graph neural network functions parameterized by ∅ . Since the input of the GVAE is the output of the Graph SAGE Encoder, ${GNNEncoder}_{\tilde{\emptyset }}$ contains a single graph convolutional layer, and ${GNNEncoder}_{\tilde{\emptyset }}^{\prime }$ contains a graph convolutional layer followed by a normalization layer, as shown in Figure 1. The graph convolutional layer in ${GNNEncoder}_{\tilde{\emptyset }}$ can effectively aggregate information from neighboring nodes in the graph, leveraging the graph structure to enhance the latent representation. The normalization layer in ${GNNEncoder}_{\tilde{\emptyset }}^{\prime }$ helps to stabilize the variance calculation, which is beneficial for the training of the GVAE model.

After passing through the double variational autoencoders composed of VAEs and GVAEs, the encoding result Z from VAEs and $\tilde{Z}$ from GVAEs are obtained. The encoding result Z of VAEs captures the latent probability distribution information of the initial input data, highlighting the internal patterns and uncertainties within the data. It provides a probabilistic understanding of the data, which can be crucial for uncovering hidden relationships. The encoding result $\tilde{Z}$ from GVAEs further delves into the encoding results processed by the graph SAGE encoder from the perspective of the graph structure. It extracts latent features that are more sensitive to the graph structure, enabling a more comprehensive understanding of the relationships between nodes in the microbe-disease graph. These graph-structure-aware latent features are highly relevant for accurate MDA prediction, as they can better represent the complex topological and relational characteristics of the graph.

### 2.4 Multi-source information fusion

In the realm of MDA prediction, different encoders exhibit distinct advantages and focal points during the data-processing phase. The encoding result Z produced by VAEs captures the latent probability distribution information inherent in the initial input data. This is particularly significant as it accentuates the internal patterns and uncertainties within the data. In the context of MDA, where the data can be influenced by a plethora of factors such as environmental variables, genetic diversities, and experimental inaccuracies, the ability of VAEs to model the data in a latent space provides a probabilistic understanding. This understanding is crucial for uncovering hidden relationships between microbes and diseases, as it can assist in generating new data points similar to the original ones, which may be beneficial for predicting novel associations.

On the other hand, the encoding result $\tilde{Z}$ obtained from GVAEs delves deeper into the encoding results processed by the Graph SAGE Encoder from the perspective of the graph structure. GVAEs are designed to handle graph-structured data within a variational framework. The node embeddings from the graph SAGE encoder already encapsulate local and global structural information of the graph in a feature space. GVAEs, by taking into account graph-specific properties like node connectivity (which determines the degree of connection between different nodes in the graph) and neighborhood relationships (describing the immediate and extended surroundings of each node), can extract more refined and graph-aware latent representations. These representations are of utmost importance for accurately predicting MDAs, as they can better capture the complex topological and relational information present in the graph.

The output $\tilde{X}$ of the graph SAGE encoder itself contains valuable local and global structural information of the nodes. This information is a result of the graph SAGE encoder’s unique approach of learning node representations through sampling and aggregating the neighbors of nodes, which endows it with high efficiency and scalability when dealing with microbe-disease graph data.

Concatenating these three distinct sources of information, namely, Z , $\tilde{Z}$ , and $\tilde{X}$ , enables the integration of information at different levels and from different perspectives. The process of concatenation allows for a more comprehensive representation of the microbe-disease relationships. By combining the probabilistic information from VAEs, the graph-structure-aware information from GVAEs, and the structural information from the graph SAGE encoder, the new node features can better capture the complex patterns in the microbe-disease relationships. This comprehensive representation provides more powerful feature support for subsequent analysis and prediction tasks in the context of MDA.

Given the VAE result Z , the GVAE result $\tilde{Z}$ , and the graph SAGE encoder result $\tilde{X}$ , they are fused according to Equation 12:

$$\hat{X}=\left[Z,\tilde{Z},\tilde{X}\right]$$ (12)

As a consequence, the fused microbe–disease graph can be denoted as $\tilde{G}=<V,E,\hat{X}>$ . This fused graph, with its enriched feature representation, has the potential to improve the performance of MDA prediction models by providing a more complete and accurate description of the relationships between microbes and diseases.

### 2.5 Microbe–disease association predictor

Upon the construction of the fused microbe-disease graph, the predictor focuses on the embedding vectors of a particular microbe node ${\hat{x}}_{m}$ and a disease node ${\hat{x}}_{d}$ derived from this graph. In the domain of graph-based prediction models, many graph auto-Encoder models, which are likely the underlying framework for the predictor in this study, typically utilize concatenation or inner-product calculations to carry out decoding tasks (Tan et al., 2023). However, considering the relatively small scale of the microbe-disease data, in order to alleviate the complexity for the subsequent neural network to learn the relationship between ${\hat{x}}_{m}$ and ${\hat{x}}_{d}$ , the Hadamard product is adopted to integrate the outputs of ${\hat{x}}_{m}$ and ${\hat{x}}_{d}$ . Mathematically, this operation is expressed by Equation 13:

$$\hat{z}={\hat{x}}_{m}⊙{\hat{x}}_{d}$$ (13)

Where the symbol ⊙ represents the Hadamard product operation. This element-wise multiplication effectively combines the features of the microbe and disease nodes at each corresponding dimension, resulting in a new vector $\hat{z}$ that encapsulates the joint information of both nodes.

Subsequently, after the Hadamard product operation, the resulting vector $\hat{z}$ is then fed into a subsequent neural network layer for predicting the association. This process can be represented by Equation 14:

$$s_{md}={DNNPredictor}_{\hat{\emptyset }}\left(\hat{z}\right)$$ (14)

Here, DNNPredictor consists of multiple linear layers and activation layers, all parameterized by $\hat{\emptyset }$ . The output $s_{md}$ represents the predicted association score for the microbe *m* and disease *d* pair. This score serves as an indication of the likelihood of an association between the two entities, with higher values suggesting a stronger potential association.

### 2.6 Optimization goals

The training of the model encompasses two primary types of loss functions: the Kullback-Leibler (KL) divergence loss and the predictor loss.

The KL divergence is a well-established metric for quantifying the difference between two probability distributions. In the context of the VAEs and GVAEs employed in the DVAMDA method, the KL divergence plays a crucial role in ensuring that the learned approximate posterior distribution of the latent variables closely aligns with a predefined prior distribution. By minimizing the KL divergence, the model is regularized, which in turn makes the latent space more semantically meaningful and interpretable. This regularization effect helps prevent overfitting and encourages the model to learn more generalizable patterns in the data.

Specifically, the KL divergence losses for VAEs and GVAEs are defined by Equations 15, 16:

$$KL\left(q_{\emptyset }\left(Z|X\right)\|p\left(Z\right)\right)=\frac{1}{2}\sum_{i=1}^{D}\left(\sigma_{i}^{2}+\mu_{i}^{2}-\log\left(\sigma_{i}^{2}\right)-1\right)$$ (15)

$$KL({\tilde{q}}_{\tilde{\emptyset }}\left(\tilde{Z}\left|\tilde{G})\right||\tilde{p}\left(\tilde{Z}\right)\right)=\frac{1}{2}\sum_{i=1}^{D}\left({\tilde{\sigma }}_{i}^{2}+{\tilde{\mu }}_{i}^{2}-\log\left({\tilde{\sigma }}_{i}^{2}\right)-1\right)$$ (16)

Where D denotes the dimension of the latent space. These equations quantify the dissimilarity between the learned posterior distribution and the prior distribution, guiding the model to learn a latent representation that adheres to the assumed prior characteristics.

The predictor loss, on the other hand, is intricately related to the MDA predictor within the DVAMDA method. Its fundamental purpose is to measure the disparity between the predicted associations and the actual known associations present in the training data. By minimizing this loss during the training process, the model is optimized to make more accurate predictions of MDAs.

Let $s_{md}$ represent the predicted probability of an association between microbe *m* and disease *d*, and $y_{md}$ denote the true label, where $y_{md}=1$ indicates an existing association and $y_{md}=0$ indicates no association. The binary cross-entropy loss is utilized to evaluate the predictor loss for this microbe - disease pair. Mathematically, it is defined by Equation 17:

$$L_{md}=-y_{md}\log\left(s_{md}\right)-\left(1-y_{md}\right)\log\left(1-s_{md}\right)$$ (17)

This loss function penalizes the model more severely for incorrect predictions, with the logarithm function amplifying the cost of misclassifications. The total predictor loss $L_{pred}$ over all microbe-disease pairs in the training set *S* is computed by Equation 18:

$$L_{pred}=\frac{1}{\left|S\right|}\sum_{\left(m,d\right)\in S}L_{md}$$ (18)

This average over all pairs in the training set provides a comprehensive measure of the model’s prediction error with respect to the known associations.

Consequently, the overarching optimization objective of the model is formulated by Equation 19:

$$L=L_{pred}-{\beta_{\emptyset }L}_{KL\emptyset }-{\beta_{\tilde{\emptyset }}L}_{KL\tilde{\emptyset }}$$ (19)

This objective function balances the trade-off between minimizing the predictor loss (to improve prediction accuracy) and minimizing the KL divergence losses of the VAEs and GVAEs (to ensure the quality and interpretability of the latent space). The hyperparameters $\beta_{\emptyset }$ and $\beta_{\tilde{\emptyset }}$ control the relative importance of the KL divergence losses in the overall optimization process, allowing for fine-tuning of the model’s performance based on the characteristics of the microbe-disease data.

## 3 Results

### 3.1 Experimental setting

In this experimental investigation, two datasets, namely, HMDAD and Disbiome, as presented in Table 1, were utilized to validate the proposed DVAMDA. To comprehensively evaluate the performance of the DVAMDA, a five-fold cross-validation technique was employed. In the five-fold cross-validation framework, the set of known MDAs was randomly partitioned into five non-overlapping subsets. For each iteration of model training, five of these subsets were designated as the training set, while the remaining one subset was used as the test set. This process was iterated five times, ensuring that every subset had the opportunity to serve as the test set. This approach helps to provide a more accurate and robust assessment of the model’s prediction ability, as it exposes the model to different combinations of training and test data. Given the scarcity of negative samples in the MDA data, a specific strategy was implemented to address this issue. An equal number of unknown associations as the number of known associations were selected to serve as negative samples. This balanced sampling method is crucial for obtaining reliable evaluation metrics, as it ensures that the model is not biased towards the more abundant positive or negative samples.

To quantitatively assess the performance of the MDA predictions made by the DVAMDA model, several well-established evaluation metrics were utilized. These metrics include the area under the ROC curve (AUC), which measures the model’s ability to distinguish between positive and negative samples across all possible classification thresholds. A higher AUC value indicates better discrimination power. The area under the precision-recall curve (AUPR) was also employed. AUPR focuses on the trade-off between precision (the proportion of true positives among the predicted positives) and recall (the proportion of true positives that are correctly identified). In addition, accuracy (ACC), which represents the proportion of correct predictions (both positive and negative) out of the total number of predictions, was calculated. Precision (PRE), defined as the ratio of true positives to the sum of true positives and false positives, and the F1-score (F1), which is the harmonic mean of precision and recall, were also used. These metrics together provide a comprehensive evaluation of the model’s performance in terms of prediction accuracy, precision, recall, and the ability to handle imbalanced data in the context of MDA prediction.

### 3.2 Performance evaluation

To comprehensively assess the generalization capacity of the DVAMDA model and analyze its performance variances across different training and testing datasets, the outcomes of the five-fold cross-validation were meticulously examined and are presented in Tables 2, 3.

**TABLE 2:** Results of 5-fold cross-validation on HMDAD database.

Index	AUC	AUPR	ACC	PRE	F1
1	0.9515	0.9579	0.8951	0.9451	0.8881
2	0.9736	0.9776	0.9284	0.9321	0.9278
3	0.9448	0.9409	0.8703	0.8752	0.9120
4	0.9641	0.9724	0.9277	0.9055	0.9077
5	0.9684	0.9576	0.9077	0.8757	0.9177
Average	0.9605	0.9613	0.9058	0.9067	0.9107

**TABLE 3:** Results of 5-fold cross-validation on Disbiome database.

Index	AUC	AUPR	ACC	PRE	F1
1	0.9568	0.9471	0.8811	0.8824	0.8808
2	0.9521	0.9349	0.8792	0.8891	0.8776
3	0.9420	0.9329	0.8683	0.8624	0.8693
4	0.9426	0.9273	0.8616	0.8625	0.8613
5	0.9454	0.9338	0.8638	0.8675	0.8630
Average	0.9478	0.9352	0.8708	0.8728	0.8704

Table 2 details the five-fold cross-validation results obtained on the HMDAD database. The AUC serves as a crucial metric for evaluating the model’s ability to distinguish between positive and negative classes across all possible classification thresholds. For the five folds, the AUC values are 0.9515, 0.9736, 0.9448, 0.9641, and 0.9684 respectively, with an average value of 0.9605. A high average AUC value indicates that the model exhibits excellent discrimination ability in differentiating between positive and negative samples within the HMDAD dataset. The AUPR is another significant metric. For each fold on the HMDAD database, the AUPR values are 0.9579, 0.9776, 0.9409, 0.9724, and 0.9576, averaging 0.9613. This high average AUPR value implies that the model performs quite well in optimizing the precision-recall trade-off within the context of the HMDAD dataset. The fold-specific ACC values are 0.8951, 0.9284, 0.8703, 0.9277, and 0.9077, with an average of 0.9058. This relatively high average accuracy demonstrates that the model has a relatively high overall prediction accuracy when tested on the HMDAD database. The PRE values for the five folds are 0.9451, 0.9321, 0.8752, 0.9055, and 0.8757, with an average of 0.9067. This average value suggests that the model has a considerable proportion of correctly predicted positive samples in the HMDAD database. For each fold on the HMDAD database, the F1 values are 0.8881, 0.9278, 0.9120, 0.9077, and 0.9177, with an average of 0.9107. This average F1 indicates that the model achieves a relatively balanced performance in terms of precision and recall on the HMDAD database.

Table 3 shows the 5-fold cross-validation results on the DISBIOME database. The AUC values for the five folds are 0.9568, 0.9521, 0.9420, 0.9426, and 0.9454, with an average of 0.9478. The AUPR values are 0.9471, 0.9349, 0.9329, 0.9273, and 0.9338, averaging 0.9352. The ACC values are 0.8811, 0.8792, 0.8683, 0.8616, and 0.8638, with an average of 0.8708. The PRE values are 0.8824, 0.8891, 0.8624, 0.8625, and 0.8675, with an average of 0.8728. The F1 values are 0.8808, 0.8776, 0.8693, 0.8613, and 0.8630, with an average of 0.8704.

Table 4 presents the 5-fold cross-validation results of the DVAMDA model on the MMDA database, which integrates data from multiple sources to form a comprehensive dataset with 1177 microbes, 134 diseases, and 4499 associations. The model achieves an average AUC of 0.9517 and AUPR of 0.9529, demonstrating strong discriminative ability in distinguishing positive and negative associations across all folds. The average ACC is 0.7983, reflecting a relatively high overall prediction correctness despite the dataset’s complexity. Notably, the model excels in PRE, with an average of 0.9712, indicating a high reliability of predicted positive associations. The F1-score, averaging 0.7513, balances precision and recall, showcasing the model’s capacity to handle imbalanced data. Overall, DVAMDA performs robustly on MMDA, highlighting its effectiveness in capturing complex microbe-disease relationships even in large-scale, heterogeneous networks.

**TABLE 4:** Results of 5-fold cross-validation on MMDA database.

Index	AUC	AUPR	ACC	PRE	F1
1	0.9577	0.9572	0.8171	0.9755	0.7789
2	0.9408	0.9485	0.8176	0.9773	0.7799
3	0.9416	0.9447	0.7874	0.9681	0.7353
4	0.9546	0.9531	0.7851	0.9619	0.7334
5	0.9638	0.9608	0.7842	0.9733	0.7290
Average	0.9517	0.9529	0.7983	0.9712	0.7513

In summary, the performance evaluation based on the five-fold cross-validation on both the HMDAD and Disbiome databases reveals that the DVAMDA model shows strong performance on these two datasets.

### 3.3 Comparisons with existing methods

To comprehensively evaluate the efficacy of the newly-proposed DVAMDA model, which ingeniously integrates double variational auto-encoders and multi-information fusion techniques, a rigorous comparative analysis is conducted against a selection of prominent models that are widely utilized in MDA tasks. The comparison models incorporated in this study are elaborated upon in detail as follows:

BiRWHDMA represents a bi-random wandering-based approach that operates within a heterogeneous network framework (Zou et al., 2017).
MVGCNMDA is a multi-view graph augmented convolutional network model, capitalizes on the power of graph convolutional networks (Hua et al., 2022).
RNMFMDA focuses on the crucial aspect of reliable negative sample selection which s based on the concepts of random wandering with restart and positive unmarked learning (Peng Lihong et al., 2020).
GATMDA combines graph attention networks and inductive matrix complementation (Long et al., 2021).
NTSHMDA is a novel computational model that integrates network topological similarity through random walk algorithms (Luo and Long, 2020).
MDAKRLS is founded on Kronecker regularized least squares with different Kronecker similarities (Cheng et al., 2023).
MNNMDA applies a matrix nuclear norm method to known microbe and disease data (Liu Haiyan et al., 2023).
CMFHMDA decomposes the MDA matrix into lower-dimensional matrices, representing the latent factors of microbes and diseases (Ma and Liu, 2022).
NCPLP is built upon the principles of network consistency projection and label propagation (Yin et al., 2022).
MSLINE integrates multiple similarities and large-scale information network embedding based on known associations (Wang et al., 2022).
DSAERF introduces RF as the final classifier model because it can work effectively on large datasets and have high training speed and accuracy (Chen Jing et al., 2024).
LRLSHMDA developes a semi-supervised computational model by introducing Gaussian interaction profile kernel similarity calculation and Laplacian regularized least squares classifier (Wang et al., 2017).
ABHMDA developes a model of Adaptive Boosting by calculating the relation probability of disease-microbe pair using a strong classifier (Peng et al., 2018).

**FIGURE 2:**
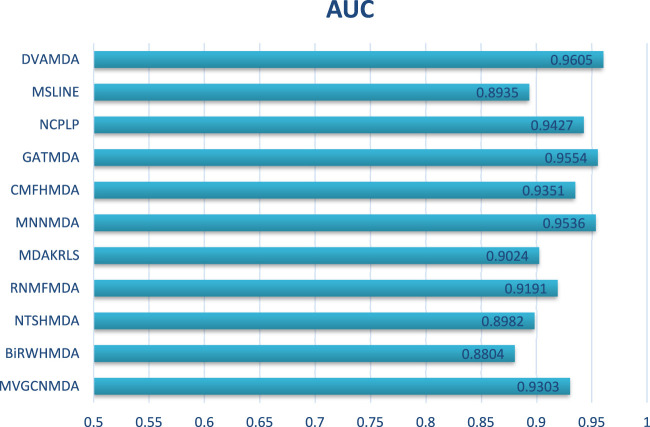
AUCs of the performance comparison on HMDAD database.

**FIGURE 3:**
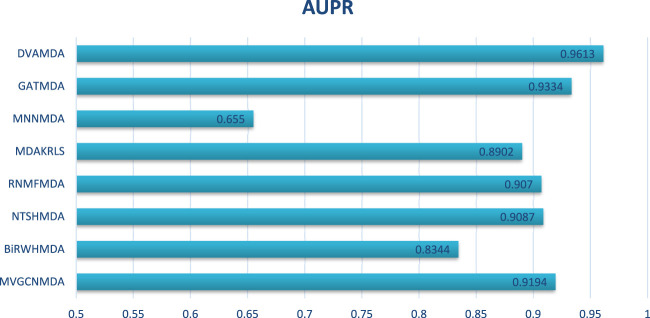
AUPRs of the performance comparison on HMDAD database.

The HMDAD database, containing 292 microbes, 39 diseases, and 450 experimentally validated associations, serves as a benchmark for evaluating microbe-disease association prediction models. Figures 2, 3 present the area under the receiver operating characteristic curve (AUC) and the area under the precision-recall curve (AUPR), respectively.

The proposed DVAMDA achieved an average AUC of 0.9605 and an AUPR of 0.9613, significantly outperforming comparative methods. For instance, MVGCNMDA, a multi-view graph convolutional model, achieved AUC and AUPR values of 0.9303 and 0.9194, respectively—highlighting DVAMDA’s advantage in fusing latent probability distributions from raw data with graph-structured features via dual variational autoencoders. Traditional approaches like BiRWHMDA, relying on bi-random walks in heterogeneous networks, lagged notably with AUC = 0.8804 and AUPR = 0.8344, demonstrating the limitations of shallow topological similarity measures in capturing complex associations. These results indicate that DVAMDA’s multi-information fusion strategy enhances model generalization even in small-scale, sparse datasets, enabling precise identification of disease-related microbes.

**FIGURE 4:**
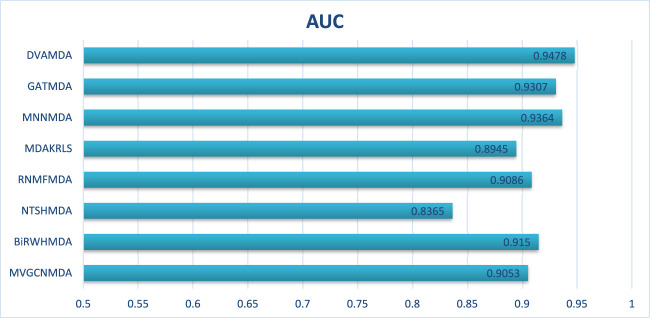
AUCs of the performance comparison on Disbiome database.

**FIGURE 5:**
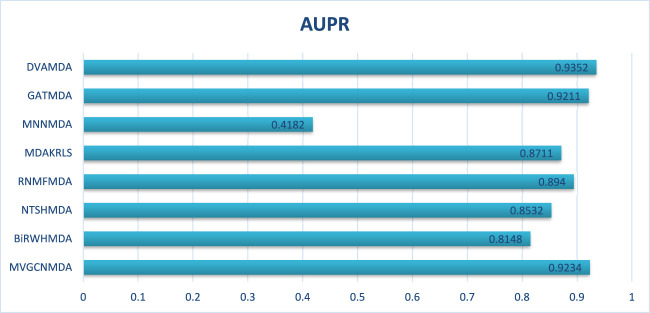
AUPRs of the performance comparison on Disbiome database.

The Disbiome database encompasses 1052 microbes, 218 diseases, and 4351 associations, presenting challenges due to its large scale and heterogeneous structure. As shown in Figures 4, 5, DVAMDA maintained robust performance with an average AUC of 0.9478 and an AUPR of 0.9352, surpassing competing methods. Compared to MVGCNMDA—achieving AUC = 0.9053 and AUPR = 0.9234—DVAMDA’s Graph SAGE encoder, combined with graph variational autoencoders, more effectively integrates local neighborhood aggregation and global structural information. This improves representation learning for nodes with sparse connections. Methods like BiRWHMDA, which exhibit sensitivity to noisy data and dependence on heuristic similarity metrics, showed reduced performance (AUC = 0.9150, AUPR = 0.8148). The results emphasize DVAMDA’s capability to model graph-specific properties such as node connectivity and neighborhood relationships, ensuring stable performance in medium-scale networks and providing a reliable framework for mining disease-associated microbiomes.

**FIGURE 6:**
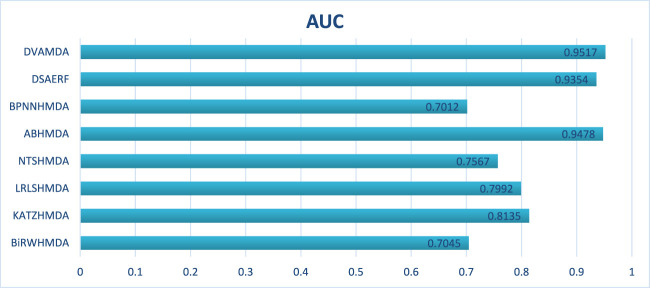
AUCs of the performance comparison on MMDA database.

**FIGURE 7:**
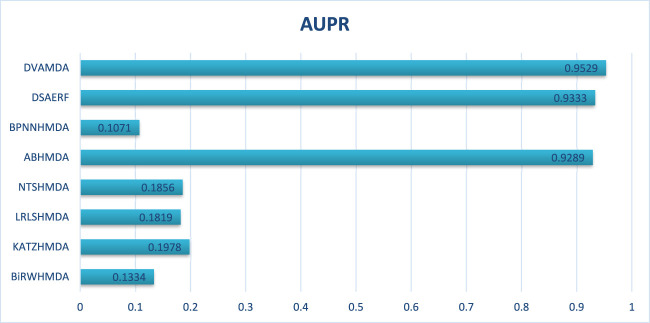
AUPRs of the performance comparison on MMDA database.

The MMDA database, integrating 1177 microbes, 134 diseases, and 4499 associations from multiple sources, serves as a critical testbed for large-scale, complex network scenarios. Figures 6, 7 show that DVAMDA achieved state-of-the-art results with an AUC of 0.9517 and an AUPR of 0.9529, outperforming eight comparative methods. Models such as ABHMDA and DSAERF-relying on ensemble learning or deep autoencoders—lagged behind due to limitations in fusing multi-source features and extracting hierarchical graph structures, achieving AUC values of 0.9478 and 0.9354, respectively. Traditional approaches like BiRWHMDA and NTSHMDA exhibited substantial gaps, with AUC values of 0.7045 and 0.7567, respectively. This highlights the inefficiency of single-similarity metrics in heterogeneous networks. DVAMDA’s superiority stems from its hierarchical framework: the Graph SAGE encoder captures fundamental structural priors, while dual variational autoencoders refine these features with probabilistic and graph-specific information. This synergy enables robust modeling of non-linear relationships and mitigates data sparsity, establishing DVAMDA as a scalable solution for predicting microbe-disease associations in large-scale networks and supporting evidence-based traditional medicine discovery.

In conclusion, through the comparison presented in Figures 2–7, it is evident that our DVAMDA model, with its innovative use of double variational autoencoders and multi-information fusion, demonstrates superior performance in predicting MDAs compared to existing models, whether on the HMDAD database or the Disbiome database.

### 3.4 Ablation study

To validate the role of each core component in the DVAMDA model, an ablation study was conducted by removing key modules, replacing graph encoding methods, and adjusting the link prediction strategy. The performance results on the HMDAD, Disbiome, and MMDA datasets (Table 5) reveal the critical contributions of the graph neural network encoder, dual variational autoencoders (DVA), and multi-information fusion strategy in capturing complex microbe-disease associations.

**TABLE 5:** Results of ablation study.

Method	HMDAD		Disbiome		MMDA	
	AUC	AUPR	AUC	AUPR	AUC	AUPR
Impact of Key Components Removal						
SAGE + DVA + Predictor(DVAMDA)	0.9605	0.9613	0.9478	0.9352	0.9517	0.9529
SAGE + DVA + degree + Predictor	0.9423	0.9498	0.9302	0.9134	0.9342	0.9253
DVA + Predictor	0.9476	0.9511	0.9333	0.9175	0.9066	0.8608
GVA + Predictor	0.9402	0.9471	0.9335	0.9176	0.8765	0.8823
GVA + degree + Predictor	0.9396	0.9464	0.9307	0.9196	0.8853	0.8333
SAGE + Predictor	0.9513	0.9555	0.9205	0.9112	0.9020	0.9262
Effects of Alternative Graph Encoding Methods						
Node2Vec + Predictor	0.5990	0.7173	0.8641	0.8774	0.8160	0.8532
LINE + Predictor	0.5986	0.7164	0.8663	0.8808	0.8261	0.8632
GCN + Predictor	0.9370	0.9433	0.9275	0.9092	0.8902	0.8858
Influence of Link Prediction Strategies						
AdamicAdarMDA	0.5458	0.5915	0.7311	0.7318	0.4397	0.4933
MGAE	0.9031	0.8600	0.8850	0.8408	0.9258	0.9195
HLGNN	0.8258	0.8305	0.8035	0.8090	0.9442	0.9433

#### 3.4.1 Impact of key components removal

Six configurations were tested to evaluate the necessity of core components, with each configuration integrating descriptions of setups, performance, and mechanistic insights through coherent narratives:

##### 3.4.1.1 Full model (SAGE + DVA + Predictor)

As the baseline setup, this configuration combines the Graph SAGE encoder, dual variational autoencoders (DVA, including VAEs for modeling raw data distributions and GVAEs for extracting graph-structured features), and a Hadamard product-based deep neural network (DNN) predictor. It achieved state-of-the-art performance with AUC values of 0.9605, 0.9478, and 0.9517, and AUPR values of 0.9613, 0.9352, and 0.9529 on the HMDAD, Disbiome, and MMDA datasets, respectively. All metrics were optimal, demonstrating the synergy between Graph SAGE’s extraction of nodal structural features, DVA’s fusion of latent probability distributions from raw data and graph structures, and the predictor’s capture of non-linear associations to form a comprehensive feature learning and prediction framework.

##### 3.4.1.2 Removing graph SAGE encoder (DVA + Predictor)

This configuration excludes the Graph SAGE encoder, directly feeding raw node features (such as microbial functional similarities and disease semantic features) into DVA for feature transformation before prediction. Performance showed notable declines: the HMDAD AUC dropped to 0.9476, a decrease of 1.3%; the Disbiome AUC fell to 0.9333, a decrease of 1.5%; and the MMDA AUC decreased to 0.9066, a decrease of 4.7%. This is attributed to the critical role of Graph SAGE’s neighborhood aggregation in extracting local and global structural priors; without it, DVA processes raw features lacking structural context, reducing the richness of latent representations.

##### 3.4.1.3 Removing dual variational autoencoders (SAGE + Predictor)

Retaining the Graph SAGE encoder but removing DVA, this configuration uses the encoder’s output features directly for prediction via the Hadamard product and DNN. Moderate performance reductions were observed: the HMDAD AUC was 0.9513, a decrease of 0.9% compared to the full model; the Disbiome AUC was 0.9205, a decrease of 2.7%; and the MMDA AUC was 0.9020, a decrease of 5.0%. The mechanism behind this is that without DVA, the model relies solely on Graph SAGE’s structural features, missing the latent probability distributions of raw data (a function of VAEs) and graph-specific properties (a function of GVAE). This leads to a bias toward structural information, ignoring deep associations in non-structural attributes such as microbial functional similarities.

##### 3.4.1.4 Retaining only graph variational autoencoders (GVA + Predictor)

By removing both the VAE and Graph SAGE, this configuration uses GVAEs to process dimension-aligned raw node features and extract graph-structured latent features (such as nodal connectivity and neighborhood relationships) for prediction. Substantial performance drops occurred: the HMDAD AUC decreased to 0.9402, a decrease of 2.1%; the Disbiome AUC fell to 0.9335, a decrease of 1.5%; and the MMDA AUC dropped to 0.8765, a decrease of 7.5%. Although GVAEs capture deep graph-structural features, the absence of VAE’s modeling of raw data distributions (e.g., microbial functions, disease semantics) restricts feature representations to structural information, failing to integrate non-structural latent associations that are critical for microbe-disease association (MDA) prediction.

##### 3.4.1.5 GVA with node degree features (GVA + degree + Predictor)

This configuration adds node degree, a handcrafted topological feature measuring nodal connectivity, to GVA outputs for feature fusion before prediction. Specifically, when constructing the hypergraph G=<V,E,X> , the degree encoder processes node degrees to capture structural context. It is integrated into the feature-encoding stage, where it helps refine the initial node feature matrix X. For the detailed process of using the degree encoder, please refer to Reference (Tan et al., 2023). By encoding degree-related structural information, it aids in better representing microbes and diseases within the hypergraph framework. The performance showed marginal declines or plateaus: the HMDAD AUC decreased by 0.0006, the Disbiome AUC decreased by 0.0028, and the MMDA AUPR experienced a significant decrease of 4.9%. This indicates that node degree, as a shallow topological metric, is redundant with the deep graph-structural features learned by GVAEs and may introduce noise. GVAEs already distill core neighborhood relationships through variational inference, making handcrafted features unnecessary and potentially detrimental to model efficiency.

##### 3.4.1.6 Full model with node degree features (SAGE + DVA + degree + Predictor)

By concatenating node degree with the outputs from Graph SAGE, VAE, and GVAE for feature fusion in the full model, notable degradations were observed: the HMDAD AUC was 0.9423, a decrease of 1.9% compared to the full model; the Disbiome AUC was 0.9302, a decrease of 1.8%; and the MMDA AUC was 0.9342, a decrease of 1.8%. The reason lies in the fact that the full model’s multi-information fusion already integrates structural, probabilistic, and graph-specific features. Adding node degree introduces low-value information that disrupts the consistency of deep-learned features, confirming that end-to-end learning outperforms manual feature engineering.

#### 3.4.2 Effects of alternative graph encoding methods

To validate the irreplaceability of the Graph SAGE encoder together with DVA, three graph embedding techniques were compared, such as Node2Vec (Grover et al., 2016), large-scale information network embedding (LINE) (Tang et al., 2015), and graph convolutional network (GCN), each differing in their approach to structural encoding and scalability in sparse graphs:

##### 3.4.2.1 Node2Vec

Node2Vec generates node embeddings via biased random walks, balancing local (BFS) and global (DFS) structural exploration to capture multi-scale relationships. However, in microbe-disease networks—characterized by sparsity and complex long-range associations-its performance was suboptimal, achieving AUC ≤0.60 on HMDAD and ≤0.87 on Disbiome. The method’s reliance on random walks led to insufficient exploration of global structures in sparse graphs, limiting its ability to model high-order nodal relationships essential for accurate MDA prediction.

##### 3.4.2.2 LINE

LINE explicitly models first-order (direct edges) and second-order (shared neighbors) similarities, making it efficient for large sparse graphs. However, it underperformed in this task, with AUC metrics comparable to Node2Vec (HMDAD ≤0.60, Disbiome ≤0.87). The limitation stems from its focus on shallow neighborhood statistics, which fail to capture the complex topological and functional associations inherent in microbe-disease networks. Specifically, LINE’s neglect of higher-order structural dependencies (e.g., community-level interactions) resulted in impoverished feature representations.

##### 3.4.2.3 GCN

GCN aggregates features from nodes and their neighbors via graph convolution, effectively capturing local structural information. It achieved moderate performance (HMDAD AUC = 0.9370) but still lagged behind Graph SAGE. The shortfall arises from GCN’s reliance on full adjacency matrices, which are inefficient and less robust for sparse graphs like MDAs. In contrast, Graph SAGE’s neighborhood sampling strategy reduces computational complexity while preserving critical structural information, making it better suited to the sparse and heterogeneous nature of microbe-disease networks.

#### 3.4.3 Influence of link prediction strategies

To assess the effectiveness of the proposed link prediction strategy in DVAMDA, it is compared with three alternative methods: (Adamic and Adar, 2003), masked graph autoencoder (MGAE) (Tan et al., 2023), and Heuristic Learning Graph Neural Network (HLGNN) (Zhang et al., 2024). These approaches represent distinct paradigms in link prediction, ranging from heuristic similarity measures to graph-based deep learning techniques, allowing a comprehensive evaluation of DVAMDA’s prediction mechanism.

##### 3.4.3.1 AdamicAdarMDA

AdamicAdarMDA employs the Adamic-Adar index, a heuristic metric that assigns higher weights to rare common neighbors between network nodes, to measure the similarity between microbes and diseases. This method is based on the assumption that nodes sharing fewer common neighbors are more likely to be associated. However, as shown in Table 5, its performance was suboptimal across all datasets. It achieved AUC values of 0.5458 on HMDAD, 0.7311 on Disbiome, and 0.4397 on MMDA. The limitations arise from its dependence on shallow topological features-specifically, first-order neighbor statistics-which fail to capture the complex nonlinear relationships and multi-scale interactions inherent in microbe-disease associations. In sparse networks like MDAs, where direct connections are scarce, heuristic measures often miss latent associations beyond simple co-occurrence patterns, leading to poor predictive performance.

##### 3.4.3.2 MGAE

MGAE is a graph autoencoder framework that masks node features or edges during training to reconstruct the graph structure, aiming to learn robust latent representations. While it utilizes deep learning for feature extraction, its performance in MDA prediction was moderate. It achieved AUC values of 0.9031 on HMDAD, 0.8850 on Disbiome, and 0.9258 on MMDA. The shortfall stems from its focus on structural reconstruction rather than integrating diverse feature types, such as microbial functional similarities and disease semantic features. Unlike DVAMDA, which fuses probabilistic, structural, and graph-specific information, MGAE relies primarily on graph topological data, neglecting non-structural attributes that are critical for capturing the biological context of MDAs. This limitation becomes more pronounced in heterogeneous networks where multi-source information is essential for accurate predictions.

##### 3.4.3.3 HLGNN

HLGNN integrates heuristic rules into graph neural networks to guide link prediction, balancing data-driven learning with prior knowledge. It demonstrated relatively better performance on the MMDA dataset (AUC = 0.9442) but lagged behind DVAMDA on HMDAD (AUC = 0.8258) and Disbiome (AUC = 0.8035). The method’s reliance on handcrafted heuristics, such as path-based similarity metrics, restricts its ability to adapt to the nuanced patterns in microbe-disease interactions. In contrast, DVAMDA’s data-driven approach—using the Hadamard product to fuse multi-level features and a deep neural network (DNN) to model nonlinear relationships—more effectively captures complex associations without explicit prior assumptions. The DNN in DVAMDA can automatically learn hierarchical representations from fused features, enabling better generalization to unseen MDAs.

### 3.5 Case study

To validate the practical utility of DVAMDA in uncovering biologically meaningful associations, we conducted case studies on three diseases—type 1 diabetes (T1D), irritable bowel syndrome (IBS), and liver cirrhosis-using the HMDAD and Disbiome datasets. For each disease, all known microbe-disease associations were excluded from the training data, and the pre-trained model was employed to predict potential associations, with the top 10 ranked microbes further validated against existing literature (Tables 6–8).

**TABLE 6:** The top 10 potential microbes related to T1D identified by DVAMDA.

Rank	Microbe	PMID
1	Cronobacter	22043294
2	*Enterobacter*	22043294
3	Bacteroidetes	20613793 22043294
4	Proteobacteria	22043294
5	*Clostridium* ramosum	22043294
6	Bacteroidaceae	20613793
7	*Clostridium* innocuum	22043294
8	Desulfotomaculum	22043294
9	Bacterium B4C2-5	22043294
10	Brenneria	22043294

**TABLE 7:** The top 10 potential microbes related to IBS identified by DVAMDA.

Rank	Microbes	PMID
1	Eubacteriaceae	21741921
2	Clostridiaceae	21741921
3	Alteromonadaceae	21741921
4	Proteobacteria	21741921
5	Syntrophobacteraceae	21741921
6	Desulfovibrionaceae	21741921
7	Coprococcus	17631127
8	Dorea	21741921
9	Ruminococcus	21741921
10	Lachnospiraceae	21741921

**TABLE 8:** The top 10 potential microbes related to Liver cirrhosis identified by DVAMDA.

Rank	Microbes	PMID
1	Fusobacteria	25079328
2	Ruminococcus gnavus	25079328
3	*Streptococcus* salivarius	21741921
4	Megasphaera	25079328
5	Prevotella	25079328
6	*Clostridium*	25079328
7	Coprococcus	25079328
8	*Haemophilus* parainfluenzae	25079328
9	Firmicutes	Unconfirmed
10	Ruminococcus gnavus	25079328

Table 6 presents the top 10 microbes predicted by DVAMDA for T1D, a chronic metabolic disorder linked to gut microbiota dysbiosis. Notably, all predicted microbes are supported by literature references (PMID: 20613793, 22043294), including Cronobacter (rank 1) and *Enterobacter* (rank 2), which have been implicated in intestinal mucosal immune activation and β-cell dysfunction—key pathological pathways in T1D development. Bacteroidetes (rank 3), validated by two independent studies, highlights the model’s ability to prioritize microbes with multi-source evidence. These results demonstrate DVAMDA’s capacity to identify biologically relevant associations even in datasets with limited disease-specific training samples (HMDAD: 39 diseases, 292 microbes), showcasing its robustness in sparse microbial networks.

Table 7 focuses on IBS, a gastrointestinal disorder characterized by gut-brain axis dysfunction. The top-ranked microbes (Eubacteriaceae, Clostridiaceae, Alteromonadaceae) are all supported by PMID 21741921, which reports their role in modulating intestinal permeability and inflammation—hallmarks of IBS pathogenesis. Coprococcus (rank 7, PMID: 17631127) is associated with altered short-chain fatty acid production in IBS patients, while Ruminococcus (rank 9) and Lachnospiraceae (rank 10) are part of the core IBS microbiome identified in multiple cohort studies. The consistency between predicted results and literature evidence underscores DVAMDA’s accuracy in capturing disease-relevant microbial taxa, even in complex multi-factorial disorders.

Table 8 illustrates predictions for liver cirrhosis, a progressive liver disease often linked to gut microbiota translocation. Most predicted microbes (Fusobacteria, Ruminococcus gnavus, Prevotella) are validated by PMID 25079328, which identifies them as key players in portal hypertension and hepatic encephalopathy. *Streptococcus* salivarius (rank 3, PMID: 21741921) has been associated with bacterial translocation in liver diseases, while Firmicutes (rank 9, unconfirmed) represents a novel candidate yet to be experimentally validated. This unconfirmed prediction highlights DVAMDA’s potential to generate hypothesis-driven targets for mechanistic studies, demonstrating its value as a discovery tool for unexplored microbe-disease relationships.

These findings strongly suggest that the DVAMDA algorithm is effective in predicting candidate microbes for a given disease. It can potentially serve as a valuable tool for researchers to explore the relationships between diseases and microbes, facilitating further in-depth investigations into the underlying mechanisms and possible therapeutic interventions.

## 4 Discussion

In this study, we successfully proposed the DVAMDA model for predicting MDAs. The model constructs a complete and efficient prediction system by integrating double variational autoencoders and multi-information fusion techniques. Firstly, the graph SAGE encoder is used to preliminarily extract the local and global structural information of nodes, providing basic data for subsequent processing. Subsequently, the double variational autoencoders play a crucial role in separately extracting the latent probability distribution information from the initial input data and the graph-related specific property information from the results of the graph SAGE encoder. Then, through the information fusion strategy, these different types of information are integrated to form a more representative feature representation, providing strong support for the prediction task. Finally, the Hadamard product operation and DNN are used to predict MDAs. A large number of experimental results show that the model exhibits excellent performance in multiple evaluation metrics, effectively verifying its feasibility and superiority in MDA prediction.

A key strength of the DVAMDA model lies in its ability to translate microbial-disease associations into tangible benefits for traditional medicine discovery, addressing a critical gap in empirical drug development. By systematically predicting MDAs across diverse disease categories, the model identifies microbial taxa that serve as potential therapeutic targets, aligning with traditional medicine’s focus on holistic microbial-host interactions. For example, in the case study on type 1 diabetes, DVAMDA prioritized gut microbes such as Cronobacter and *Enterobacter*, which are linked to intestinal immune activation—a pathway relevant to diabetic pathogenesis. Such predictions can guide the screening of herbal compounds with known antimicrobial or immunomodulatory effects, facilitating the development of precision interventions.

Looking ahead, there are several promising areas for future research. Firstly, exploring more advanced graph neural network architectures could further optimize the DVAMDA model. For example, investigating novel graph convolutional network variants or attention-based graph neural network structures might enhance the model’s ability to capture complex relationships in the microbe-disease graph. This could potentially lead to more accurate predictions of MDAs. Additionally, developing more efficient feature fusion methods could be beneficial. Instead of the current concatenation-based multi-information fusion, exploring more sophisticated techniques such as weighted fusion or hierarchical fusion might better integrate the diverse information from different encoders, improving the model’s performance.

## Data Availability

The datasets presented in this study can be found in online repositories. The names of the repository/repositories and accession number(s) can be found in the article/supplementary material.

## References

[B101] Adamic L. A. Adar E. (2003). Friends and neighbors on the Web. Social Networks, 25 (3), 211–230. 10.1016/S0378-8733(03)00009-1

[B1] Chen H. Chen K. (2024). Predicting disease - associated microbes based on similarity fusion and deep learning. Briefings Bioinforma. 25 (6), bbae550. 10.1093/bib/bbae550 PMC11540060 39504483

[B2] Chen J. Tao R. Qiu Y. Yuan Q. (2024a). CMFHMDA: a prediction framework for human disease - microbe associations based on cross - domain matrix factorization. Briefings Bioinforma. 25 (6), bbae481. 10.1093/bib/bbae481 PMC11427075 39327064

[B3] Chen R. Xie G. Lin Z. Gu G. Yu Y. Yu J. (2024b). Predicting microbe-disease associations based on a linear neighborhood label propagation method with multi-order similarity fusion learning. Interdiscip. Sci. Comput. Life Sci. 16, 345–360. 10.1007/s12539-024-00607-0 38436840

[B4] Cheng E. Zhao J. Wang H. Song S. Xiong S. Sun Y. (2023). Dual network contrastive learning for predicting microbe -disease associations. IEEE/ACM Trans. Comput. Biol. Bioinforma. 20 (6), 3469–3481. 10.1109/TCBB.2022.3228617

[B5] Dai L.-Y. Wang S. Liu J.-X. Liu B.-M. Li F. Gao Y.-L. (2023). “MKGSAGE: a computational framework via multiple kernel fusion on GraphSAGE for inferring potential disease-related microbes,” in 2023 IEEE international conference on bioinformatics and biomedicine (IEEE), 648–652. 10.1109/BIBM58861.2023.10385527

[B6] Diederik P. K. Welling M. (2019). An introduction to variational autoencoders. arXiv:1906.02691 12, 307–392. 10.1561/2200000056

[B7] Fan C. Lei X. Guo L. Zhang A. (2019). Predicting the associations between microbes and diseases by integrating multiple data sources and path-based HeteSim scores. Neurocomputing 323, 76–85. 10.1016/j.neucom.2018.09.054

[B8] Grover A. Leskovec J. Kocijan V. (2016). “node2vec: scalable feature learning for networks,” in Proceedings of the 30th ACM SIGKDD conference on knowledge discovery and data mining, 855–864. 10.1145/2939672.2939754 PMC5108654 27853626

[B9] Guan J. Zhang Z. G. Liu Y. Wang M. (2022). A novel bi-directional heterogeneous network selection method for disease and microbial association prediction. BMC Bioinforma. 23, 483. 10.1186/s12859-022-04961-y PMC9664813 36376802

[B10] Hamilton W. L. Ying R. Jure L. (2017). “Inductive representation learning on large graphs,” in International conference on neural information processing systems, 1025–1035.

[B11] He L. Zou Q. Qi D. Cheng S. Wang Y. (2024). Adversarial regularized autoencoder graph neural network for microbe-disease associations prediction. Briefings Bioinforma. 25 (6), bbae584. 10.1093/bib/bbae584 PMC11554402 39528423

[B12] Hua M. Yu S. Liu T. Yang X. Wang H. (2022). MVGCNMDA: multi-view graph augmentation convolutional network for uncovering disease-related microbes. Interdiscip. Sci. Comput. Life Sci. 14, 669–682. 10.1007/s12539-022-00514-2 35428964

[B13] Janssens Y. Nielandt J. Bronselaer A. Debunne N. Verbeke F. Wynendaele E. (2018). Disbiome database: linking the microbiome to disease. BMC Microbiol. 18 (1), 50. 10.1186/s12866-018-1197-5 29866037 PMC5987391

[B14] Jiang C. Tang M. Jin S. Huang W. Liu X. (2023). KGNMDA: a knowledge graph neural network method for predicting microbe-disease associations. IEEE/ACM Trans. Comput. Biol. Bioinforma. 20 (2), 1147–1155. 10.1109/TCBB.2022.3184362 35724280

[B15] Kostic A. Gevers D. Siljander H. Vatanen T. Hyötyläinen T. Hämäläinen A. (2015). The dynamics of the human infant gut microbiome in development and in progression toward type 1 diabetes. Cell. Host Microbe 17 (2), 260–273. 10.1016/j.chom.2015.01.001 25662751 PMC4689191

[B16] Kumar A. Singh S. S. Singh K. (2020). Bhaskar BiswasLink prediction techniques, applications, and performance: a survey. Phys. A Stat. Mech. its Appl. 551 (9), 124289. 10.1016/j.physa.2020.124289

[B17] Li H. Wang Y. Zhang Z. Tan Y. Chen Z. Wang X. (2021). Identifying microbe-disease association based on a novel back-propagation neural network model. IEEE/ACM Trans. Comput. Biol. Bioinforma. 18 (6), 2502–2513. 10.1109/TCBB.2020.2986459 32305935

[B18] Liu D. Liu J. Luo Y. He Q. Deng L. (2022). MGATMDA: predicting microbe-disease associations via multi-component graph attention network. IEEE/ACM Trans. Comput. Biol. Bioinforma. 19 (6), 3578–3585. 10.1109/TCBB.2021.3116318 34587092

[B19] Liu H. Bing P. Zhang M. Tian G. Ma J. Li H. (2023a). MNNMDA: predicting human microbe-disease association via a method to minimize matrix nuclear norm. Comput. Struct. Biotechnol. J. 21, 1414–1423. 10.1016/j.csbj.2022.12.053 36824227 PMC9941872

[B20] Liu J.-X. Yin M.-M. Gao Y.-L. Shang J. Zheng C.-H. (2023b). MSF-LRR: multi-similarity information fusion through low-rank representation to predict disease-associated microbes. IEEE/ACM Trans. Comput. Biol. Bioinforma. 20 (1), 534–543. 10.1109/TCBB.2022.3146176 35085090

[B21] Liu Y. Wang S.-L. Zhang J.-F. Zhang W. Zhou S. Wen L. (2021). DMFMDA: prediction of microbe-disease associations based on deep matrix factorization using bayesian personalized ranking. IEEE/ACM Trans. Comput. Biol. Bioinforma. 18 (5), 1763–1772. 10.1109/TCBB.2020.3018138 32816678

[B22] Long Y. Luo J. Zhang Y. Xia Y. (2021). Predicting human microbe–disease associations via graph attention networks with inductive matrix completion. Briefings Bioinforma. 3, bbaa146. 10.1093/bib/bbaa146 32725163

[B23] Lu S. Liang Y. Li L. Miao R. Liao S. Zou Y. (2023). Predicting potential microbe-disease associations based on auto-encoder and graph convolution network. BMC Bioinforma. 24, 476. 10.1186/s12859-023-05611-7 PMC10722760 38097930

[B24] Luo J. Long Y. (2020). NTSHMDA: prediction of human microbe-disease association based on random walk by integrating network topological similarity. IEEE/ACM Trans. Comput. Biol. Bioinforma. 17 (4), 1341–1351. 10.1109/TCBB.2018.2883041 30489271

[B25] Ma W. Zhang L. Zeng P. Huang C. Li J. Geng B. (2017). An analysis of human microbe-disease associations. Briefings Bioinforma. 18 (1), 85–97. 10.1093/bib/bbw005 26883326

[B26] Ma Y. Liu Q. (2022). Generalized matrix factorization based on weighted hypergraph learning for microbe-drug association prediction. Comput. Biol. Med. 145, 105503. 10.1016/j.compbiomed.2022.105503 35427986

[B27] Naik A. Patwardhan I. Joshi A. (2024). CGDGMDA-Net: discovering microbe-disease and drug associations through CTGAN and graph-based deep learning. Netw. Model. Analysis Health Inf. Bioinforma. 13, 48. 10.1007/s13721-024-00484-z

[B28] Peng L. Dong Z. Liu W. Zhou L. Wang L. Zhao B. (2020a). Prioritizing human microbe-disease associations utilizing a node-information-based link propagation method. IEEE Access 8, 31341–31349. 10.1109/ACCESS.2020.2972283

[B29] Peng L. Shen L. Liao L. Liu G. Zhou L. (2020b). RNMFMDA: a microbe-disease association identification method based on reliable negative sample selection and logistic matrix factorization with neighborhood regularization. Front. Microbiol. 11, 592430. 10.3389/fmicb.2020.592430 33193260 PMC7652725

[B30] Peng L. Yin J. Zhou L. Liu M. Zhao Y. (2018). Human microbe-disease association prediction based on adaptive boosting. Front. Microbiol. 9, 2440. 10.3389/fmicb.2018.02440 30356751 PMC6189371

[B31] Peng W. Liu M. Dai W. Chen T. Fu Y. Pan Y. (2023). Multi-view feature aggregation for predicting microbe-disease association. IEEE/ACM Trans. Comput. Biol. Bioinforma. 20 (5), 2748–2758. 10.1109/TCBB.2021.3132611 34871177

[B32] Schriml L. M. Munro J. B. Schor M. Olley D. McCracken C. Felix V. (2022). The human disease ontology 2022 update. Nucleic Acids Res. 50 (D1), D1255–D1261. 10.1093/nar/gkab1063 34755882 PMC8728220

[B33] Tan Q. Liu N. Huang X. Choi S.-H. Li L. Chen R. (2023). “S2GAE: self-supervised graph autoencoders are generalizable learners with graph masking,” in ACM international conference on web search and data mining, 787–795.

[B34] Tang J. Qu M. Wang M. Zhang M. Yan J. Mei Q. (2015). “LINE: large-scale information network embedding,” in Proceedings of the 24th international conference on world wide web, 1067–1077. 10.1145/2736277.274109

[B35] Wang F. Huang Z. Chen X. Zhu Z. Wen Z. Zhao J. (2017). LRLSHMDA: laplacian regularized least squares for human microbe–disease association prediction. Sci. Rep. 7, 7601–7611. 10.1038/s41598-017-08127-2 28790448 PMC5548838

[B36] Wang J. Lu D. Wang L. Lin Y. Xu Q. (2023a). “PUNNHMDA: microbe-Disease Association prediction based on PU learning and optimized K-Nearest Neighbor algorithm,” in 2023 IEEE 11th joint international information Technology and artificial intelligence conference (ITAIC) (IEEE), 147–153. 10.1109/ITAIC58329.2023.10409078

[B37] Wang L. Wang Y. Xuan C. Zhang B. Wu H. Gao J. (2023b). Predicting potential microbe–disease associations based on multi - source features and deep learning. Briefings Bioinforma. 24 (4), bbad255–14. 10.1093/bib/bbad255 37406190

[B38] Wang S. Liu J.-X. Li F. Wang J. Gao Y.-L. (2024b). M3HOGAT: a multi-view multi-modal multi-scale high-order graph attention network for microbe-disease association prediction. IEEE J. Biomed. Health Inf. 28 (10), 6259–6267. 10.1109/JBHI.2024.3429128 39012741

[B39] Wang W. Yan Q. Liao Q. Jin X. Gong Y. Zhuo L. (2024a). Multi-scale information fusion and decoupled representation learning for robust microbe-disease interaction prediction. J. Pharm. Analysis, 101134. 10.1016/j.jpha.2024.101134 PMC12491710 41050112

[B40] Wang Y. Lei X. Lu C. Pan Y. (2022). Predicting microbe-disease association based on multiple similarities and LINE algorithm. IEEE/ACM Trans. Comput. Biol. Bioinforma. 19 (4), 2399–2408. 10.1109/TCBB.2021.3082183 34014827

[B41] Wang Y. Lei X. Pan Y. (2023c). Microbe-disease association prediction using RGCN through microbe-drug-disease network. IEEE/ACM Trans. Comput. Biol. Bioinforma. 20 (6), 3353–3362. 10.1109/TCBB.2023.3247035 37027603

[B42] Wu C. Lin B. Zhang H. Xu D. Gao R. Song R. (2025). GCNPMDA: human microbe - disease association prediction by hierarchical graph convolutional network with layer attention. Biomed. Signal Process. Control 100, 107004. 10.1016/j.bspc.2024.107004

[B43] Wu C. Xiao X. Yang C. Chen J. X. Yi J. Qiu Y. (2021). Mining microbe-disease interactions from literature via a transfer learning model. BMC Bioinforma. 22, 432. 10.1186/s12859-021-04346-7 PMC8430297 34507528

[B44] Xu D. Xu H. Zhang Y. Wang M. Chen W. Gao R. (2021). MDAKRLS: predicting human microbe-disease association based on Kronecker regularized least squares and similarities. J. Transl. Med. 19 (66), 66. 10.1186/s12967-021-02732-6 33579301 PMC7881563

[B45] Yan C. Duan G. Wu F.-X. Pan Y. Wang J. (2020). BRWMDA: predicting microbe-disease associations based on similarities and Bi-random walk on disease and microbe networks. IEEE/ACM Trans. Comput. Biol. Bioinforma. 17 (5), 1595–1604. 10.1109/TCBB.2019.2907626 30932846

[B46] Yan C. Duan G. Wu F.-X. Pan Y. Wang J. (2021). MCHMDA: predicting microbe-disease associations based on similarities and low-rank matrix completion. IEEE/ACM Trans. Comput. Biol. Bioinforma. 18 (2), 611–620. 10.1109/TCBB.2019.2926716 31295117

[B47] Yin M.-M. Gao Y.-L. Zheng C.-H. Liu J.-X. (2023). NTBiRW: a novel neighbor model based on two-tier Bi-random walk for predicting potential disease-related microbes. IEEE J. Biomed. Health Inf. 27 (3), 1644–1653. 10.1109/JBHI.2022.3229473 37022835

[B48] Yin M.-M. Liu J.-X. Gao Y.-L. Kong X.-Z. Zheng C.-H. (2022). NCPLP: a novel approach for predicting microbe-associated diseases with network consistency projection and label propagation. IEEE Trans. Cybern. 52 (6), 5079–5087. 10.1109/TCYB.2020.3026652 33119529

[B49] Yu S. Wang H. Hua M. Liang C. Sun Y. (2024). Sparse graph cascade multi - kernel fusion contrastive learning for microbe–disease association prediction. Expert Syst. Appl. 252, 124092. 10.1016/j.eswa.2024.124092

[B50] Zhang J. Wei L. Xu Z. Yao Q. (2024). “Heuristic learning with graph neural networks: a unified framework for link prediction,” in Proceedings of the 30th ACM SIGKDD conference on knowledge discovery and data mining, 4223–4231. 10.1145/3637528.3671946

[B51] Zhu H. Hongxia H. Yu L. (2024). Identification of microbe–disease signed associations via multi-scale variational graph autoencoder based on signed message propagation. BMC Biol. 22, 172. 10.1186/s12915-024-01968-0 39148051 PMC11328394

[B52] Zou S. Zhang J. Zhang Z. (2017). A novel approach for predicting microbe-disease associations by Bi-random walk on the heterogeneous network. PLoS ONE 9 (2), e0184394. 10.1371/journal.pone.0184394 PMC5589230 28880967

